# A Review of Adverse Outcomes Following Intravenous Morphine Usage for Pain Relief in Acute Coronary Syndrome

**DOI:** 10.7759/cureus.3246

**Published:** 2018-09-04

**Authors:** Annie Chen, Farimah Shariati, Tiffany Chan, David Lebowitz

**Affiliations:** 1 Emergency Medicine, University of Central Florida College of Medicine, Orlando, USA

**Keywords:** acute coronary syndrome, stemi, morphine, emergency medicine, myocardial infarction, cardiology, analgesia

## Abstract

Chest pain in acute coronary syndrome (ACS) unresponsive to nitrates is routinely treated by intravenous (IV) morphine. In the past two decades, several studies have emerged, suggesting poorer outcomes in patients receiving this treatment; however, morphine remains the drug of choice for these patients as per the American College of Cardiology/American Heart Association guidelines on ACS management. The results of various studies that examined the impact of morphine on myocardial infarct size, antiplatelet therapy absorption time, and patient mortality are discussed. There is mounting evidence suggesting that morphine may increase adverse events in ACS patients, therefore, the development of more precise criteria for IV morphine administration in ACS patients is needed.

## Introduction and background

Chest pain is easily the most common presenting complaint in acute coronary syndrome (ACS). It has been reported in as much as 94% of all patients with new-onset cardiac ischemia and is often described as a substernal pressure, squeezing, or burning sensation that may radiate to the left side of the body [1]. Administration of nitrates is usually able to provide rapid relief to pain of cardiac origin; however, in patients with severe and persistent pain or in patients with contraindications to nitrates, intravenous (IV) morphine is often used as an analgesic. Although pain severity has not been shown to have any relationship with subsequent cardiovascular complications or poorer outcomes, patient comfort is not an insignificant factor [2]. In theory, pain can even worsen the size of infarcts by stimulating the sympathetic nervous system, thereby increasing myocardial oxygen demand [3].

For many years, the American College of Cardiology (ACC)/American Heart Association (AHA) guidelines on ACS management endorsed morphine as a Class IC indication for chest discomfort unresponsive to nitrates in ST-segment elevation myocardial infarction (STEMI), non-ST-segment elevation myocardial infarction (NSTEMI), and unstable angina (UA) patients despite a lack of randomized outcome trials to evaluate its safety and efficacy or support this usage [4]. It was not until a 2005 retrospective analysis demonstrating an increased risk of mortality in patients receiving morphine was published that serious concerns were raised and the indication was eventually reduced to a Class IIb recommendation in NSTEMI and UA populations [5].

When it comes to pain management, morphine boasts several undeniably beneficial properties for pain of cardiac origin. Aside from its potent analgesic properties, morphine’s innate hemodynamic properties are ideal for counteracting a heightened sympathetic response and reducing the cardiac workload. By decreasing heart rate, blood pressure, and venous return, morphine is hypothesized to reduce myocardial oxygen demand. However, this mechanism has never been conclusively tested, and there are also concerns that potential morphine-induced respiratory depression may contribute to increased oxygen demand [3].

Furthermore, morphine has demonstrated multiple gastrointestinal adverse effects, such as nausea, vomiting, and inhibition of gut motility, which hold significant clinical implications in the treatment of ACS [3].

In recent years, several studies describing morphine’s inhibitory effects on the absorption of various oral drugs, including antiplatelet medications, such as clopidogrel and ticagrelor, have emerged. The implications of these findings are particularly concerning, as antiplatelet therapy is instrumental in the prevention of future coronary thrombotic occlusions in this population. Delays in absorption or suboptimal plasma concentrations of these drugs may contribute to or even account for the poor clinical outcomes associated with morphine in ACS [6-7].

Several studies have published their findings on the different effects of morphine use in ACS. The primary purpose of this review is to quantify the impact of IV morphine in ACS patients by evaluating the results of current studies that examined the effect of morphine on myocardial infarct sizes, interactions with antiplatelet therapy, and mortality rate when compared to patients who were not given morphine.

## Review

Gwag et al. assessed the effects of IV morphine on myocardial salvage in STEMI patients who underwent primary percutaneous coronary intervention (PCI) via cardiac magnetic resonance (CMR). This study was a prospective, single institution, observational study using a registry of patients who presented with an acute myocardial infarction and subsequently underwent CMR between January 2008 and June 2014. They focused on patients whose electrocardiogram (EKG) showed an ST-segment elevation greater than 1 mm in two or more consecutive leads or a presumably new left bundle branch block. If the patients had a previous history of myocardial infarction (MI) or revascularization, medical treatment with no primary PCI, or a symptom-to-balloon time greater than 12 hours, they were excluded from the study. Of the 515 screened patients, after inclusion and exclusion criteria were applied, 299 patients were studied [8].

All patients were given 300 mg of aspirin and 600 mg of clopidogrel as a loading dose if they had not previously taken those medications before their PCI procedure. Some patients were administered IV morphine prior to PCI, but the study does not explain how this decision was made. Using CMR, the investigators calculated a myocardial salvage index (MSI), which used the area at risk (AAR) minus the infarct size x100/ AAR. This MSI value was the primary outcome. Secondary outcomes included the AAR, myocardial infarct size, extent of microvascular obstruction (MVO), and presence of hemorrhage, as assessed by CMR and Major Adverse Cardiac Events (MACEs). MACEs include cardiac death, recurrent MI, ischemic stroke, and coronary revascularization after PCI. Of the 299 patients, 96 (32.1%) received morphine prior to primary PCI with a median dose of 5 mg. Additionally, the investigators used a post hoc propensity score-matched analysis to perform a 90 matched-pair study from the original 299 sample population [8].

The analysis showed that patients who were administered morphine prior to primary PCI had greater MSI, larger infarct sizes, and an increased occurrence of hemorrhagic infarction or MVO in the crude analysis (n=299). These same outcomes did not show a significant difference in the propensity score-matched analysis. Thus, this study concluded that IV morphine administration prior to primary PCI did not adversely affect myocardial salvage in STEMI patients [8].

While Gwag et al. attempt to produce evidence that could impact patient morbidity and mortality, flaws in study design bring into question the consistency of their data collection and, consequently, the external validity of this study [8].

Firstly, the investigators did not discuss a power analysis. Without first establishing this baseline value, Gwag et al. did not establish whether their sample size was representative of the population or had the power to accurately assess their desired outcomes. With a smaller sample and an equal number of considered variables, the propensity score-matched analysis has even less power than the crude analysis. Additionally, although the study attempts to minimize baseline differences between its subjects, it does not discuss the variance among patients who were previously taking aspirin and clopidogrel and whether this may have influenced their responses. While it is noted that the administration of IV morphine prior to PCI was not made by the individual interventionalists, there is no explanation about how the decision of IV morphine administration was made [8].

In 2015, de Waha et al. aimed to understand the impact IV morphine had on infarct size, MVO, and myocardial salvage index using CMR. They screened 291 STEMI patients who were previously enrolled in two randomized clinical trials: Leipzig immediate percutaneous coronary intervention acute myocardial infarction N-acetylcysteine (LIPSIA-N-ACC) trial and Leipzig immediate prehospital facilitated angioplasty in ST-segment myocardial infarction (LIPSIA-STEMI) trial. In this retrospective, non-randomized, double-blinded, cohort study, the included patients had symptoms <12 h and ST-segment elevation of at least 0.1 mV in two or more extremity leads or at least 0.2 mV in two or more precordial leads. The study excluded patients who had prior fibrinolysis, cardiogenic shock, or contraindications to CMR at the time of study entry. This led to a final sample size of 276 patients. One hundred and twenty-three of these patients (44.6%) received IV morphine prior to primary PCI, which was performed according to standard clinical practice in Europe. CMR measurements were performed by operators blinded to the patients’ baseline and outcome data. They used CMR evaluation software and applied a semi-automated computer-aided approach to identify infarctions [9]. Patients who received IV morphine displayed the negative outcomes of larger infarcts, a higher extent of MVOs, and significantly reduced MSIs. Even after adjustment for a detailed set of baseline characteristics, Waha et al. found that IV morphine administration continued to be an independent predictor of suboptimal reperfusion [9]. At the time of its publication, this study was the first to provide evidence of an association between IV morphine and CMR parameters for damage. The study’s maintenance of blinding investigators at all possible points in the study gives strength toward the objectivity of the data. This is further reinforced by the standardized protocol used to collect CMR parameters, including the use of a semi-automated computer-aided approach [9].

Hobl et al. participated in several trials investigating morphine’s effects on P2Y12 receptor antagonists. This particular study is a randomized, double-blind, placebo-controlled, crossover trial, specifically examining morphine’s interactions with clopidogrel, which would be quantified by plasma levels of clopidogrel active metabolite as well as its antiplatelet effects [10].

Subjects included 24 healthy individuals who received a loading dose of 600 mg clopidogrel together with placebo or 5 mg morphine intravenously. Inclusion criteria were: ≥18 years of age, non-pregnant, and the ability to comprehend the full nature and purpose of the study. Exclusion criteria were: intake of non-steroidal anti-inflammatory drugs or platelet inhibitors, known coagulation disorders, relevant impairment of renal or hepatic function, chronic infectious diseases, clinically relevant abnormal laboratory values, and contraindications for clopidogrel or morphine [10].

Each treatment was prepared by unblinded pharmacists and administered by blinded physicians. After an overnight fast, a loading dose of 600 mg clopidogrel was administered after an injection of placebo or morphine. The trials indicated that the morphine injection resulted in delayed maximal plasma concentrations of clopidogrel (Tmax: 105 vs. 83 min, p=0.025). The placebo group demonstrated pharmacokinetics consistent with a 600 mg loading dose; however, the morphine group’s pharmacokinetic profile was reflective of reduced gastrointestinal absorption of clopidogrel and more consistent with that of a 300 mg loading dose. The increased Tmax further reinforces the delayed absorption of clopidogrel. Additionally, the time required to maximally inhibit platelet aggregation was also delayed twofold (3 vs. 1.25 h, p < 0.001) and, in some cases, even up to five hours, reflecting the diminished antiplatelet effects of clopidogrel. These results suggest that the concurrent use of morphine and clopidogrel in ACS patients could prove highly detrimental and facilitate the recurrence of major adverse coronary events, including MI and death [10].

Though the results of Hobl et al. are insightful and present several important findings, they are also severely limited by a number of factors. For instance, the small enrollment size of 24 individuals restricts its external validity. Similarly, the sole inclusion of healthy subjects for this study rather than ACS patients, whose gastrointestinal absorption may be compromised, to begin with, in their acute state, further limits the applicability and external validity of these findings. Furthermore, it’s unclear from the results whether the impact of morphine on clopidogrel’s pharmacokinetics actually affect patient outcomes. For these reasons, further investigation is warranted [10].

The Influence of Morphine on Pharmacokinetics and Pharmacodynamics of Ticagrelor in Patients with Acute Myocardial Infarction (IMPRESSION) trial is a phase IV, single-center, randomized, double-blinded, placebo-controlled study that investigated the influence of morphine on the pharmacokinetics and pharmacodynamics of ticagrelor. Ticagrelor is another P2Y12 receptor antagonist considered alongside aspirin to be an essential medication in the long-term management of ACS. Due to its crucial role in attenuating platelet blockage, any hindrance to ticagrelor’s effectiveness by morphine administration may translate into an increased risk of potentially devastating thrombotic complications in this patient population [6].

The 74 patients that were enrolled in the study were assigned on a 1:1 ratio to receive either 5 mg of morphine or placebo (0.9% NaCl), which was immediately followed by 180 mg of ticagrelor. Of note, patients from both groups received a 300 mg loading dose of aspirin orally and subsequently underwent a coronary angiography assessment followed by PCI, if necessary [6].

The study population was composed of males and non-pregnant females between the ages of 18 and 80 years with an established diagnosis of STEMI or NSTEMI awaiting angiography and PCI. The study’s key exclusion criteria included unbearable chest pain, patient’s request for analgesics, prior administration of morphine during the current ACS episode, treatment with any P2Y12 receptor inhibitor within 14 days prior to enrollment, ongoing treatment with oral anticoagulant or chronic therapy with low molecular weight heparin (LMWH), active bleeding, Killip class III or IV, respiratory failure, or a history of coagulation disorders [6].

The results of the study demonstrated that when compared with the placebo, the administration of morphine resulted in a lower total exposure to both ticagrelor and its active metabolite with a difference of 55% (p=0.002) and 36% (p=0.003) at six and 12 hours respectively. Further analysis showed that the maximal plasma concentration of ticagrelor in patients receiving morphine was also delayed and reduced when compared to the placebo group. The study also found that there was a stronger antiplatelet effect in the placebo group than in morphine-treated patients. The co-administration of morphine resulted in a significantly higher platelet reactivity, reflecting an impaired antiplatelet effect by ticagrelor in patients receiving morphine when compared with the placebo group, as well as a decreased maximal plasma concentration of ticagrelor. These findings illustrated the impact exerted by morphine on the pharmacokinetics and antiplatelet action of ticagrelor in ACS patients [6].

Though the IMPRESSION trial joins the ranks of several other studies that obtained similar findings, strengthening their collective conclusion, it is simultaneously hindered by its own limitations. Its relatively small sample size of 74 prevents a thorough assessment of morphine’s clinical effects as well as the possibility of subgroup analyses. Additionally, although this study improves upon Hobl et al.’s limitations and enrolls ACS patients, the inclusion of both STEMI and NSTEMI patients will inevitably incorporate heterogeneity into the study population [6].

Parodi et al. similarly investigated the effect of morphine on platelet reactivity in a nonrandomized multicentered study. Unlike the IMPRESSION trial, Parodi et al. sought to assess platelet inhibition following a loading dose of either prasugrel or ticagrelor after stratifying for the use of morphine [7].

This study is a prospective observational trial incorporating five previous studies, including published trials by Parodi et al. and Alexopoulos et al., as well as a previously unpublished study from Catania University. Patients were stratified according to morphine use before antiplatelet agent loading dose. Residual platelet reactivity was assessed. Four hundred and ninety-six patients with a diagnosis of STEMI within 12 hours of symptom onset were enrolled. Exclusion criteria included those who were <18 years of age, had active bleeding or bleeding diathesis, had a previous transient ischemic attack or stroke, were administered ticlopidine, clopidogrel, prasugrel, ticagrelor, or glycoprotein IIb/IIIa inhibitors in the week before their ACS event, needed chronic anticoagulation therapy, had a known relevant hematologic deviation, severe liver disease, or a life expectancy of less than a year or were hemodynamically unstable [7].

Of the 496 patients, only 300 who were considered P2Y12 receptor inhibitor naive were analyzed, with 95 of those subjects treated with morphine and 205 treated without. High residual platelet reactivity (HRPR) was defined as P2Y12 reactivity units (PRU) ≥208, and patients who received morphine demonstrated higher PRU that persisted for several hours than those who did not: 182.3 PRU (95% CI, 164.2-200.3) vs. 140.3 PRU (95% CI, 128.2-152.4), with a mean difference of 42.0 PRU (95% CI, 19.8-64.1), p<0.001 [7].

Parodi et al. conclude that in patients with STEMI, morphine administration is associated with a delayed onset of action of the oral antiplatelet agents, with comparable findings in both prasugrel and ticagrelor. This study’s sample size is still not extensive enough to permit a satisfactory assessment of other potential clinical effects of morphine and the use of HRPR as a measurement of platelet inhibition is also not ideal, as HRPR is not an equivalent substitution for reduced antiplatelet effect. Despite these limitations, this study does improve on both the IMPRESSION trial and the Hobl et al. study in its significantly larger enrollment size, which bodes well for the external validity of the findings and makes important contributions to the understanding of morphine’s interactions with antiplatelet agents [7].

The Can Rapid Risk Stratification of Unstable Angina Patients Suppress ADverse Outcomes with Early Implementation of the ACC/AHA Guidelines (CRUSADE) initiative is a nonrandomized, retrospective, observational registry that seeks to evaluate interventions and outcomes in non-ST segment elevation acute coronary syndrome (NSTE-ACS) patients. The 2005 paper by Meine et al. assesses the risk of death associated with morphine use in this population and features an enrollment size of 57,039 non-ST segment elevation acute coronary syndrome (NSTE-ACS) patients, which encompasses those diagnosed with NSTEMI and UA. Individuals entered into the CRUSADE database between January 2001 and June 2003 were included in the study and data was collected anonymously during the initial hospitalization. Inclusion criteria consisted of ischemic symptoms at rest within 24 hours prior to presentation, high-risk features, such as ST-segment depression ≥0.5 mm, transient ST-segment elevation 0.5-1.0 mm lasting less than 10 minutes, and positive cardiac markers [5].

To account for non-random treatment assignment, a propensity score model was created using multivariable, generalized estimating equations to estimate the likelihood of receiving IV morphine treatment and was subsequently used to stratify the total study population into quintiles of equal size. Of the enrolled patients, 17,003 (29.8%) received morphine while 40,036 (71.2%) did not. The incidence of adverse effects is notably higher in individuals who received morphine compared to those who did not. Death increased from 4.7% to 5.5% with an unadjusted odds ratio of 1.22 and an adjusted odds ratio of 1.48, indicating that mortality may be increased by as much as 50% in morphine users. There was no indication that centers providing more morphine use were associated with suboptimal care, nor that morphine use was a marker for sicker patients. It is possible that the potent analgesic effects of morphine mask the severity of angina without alleviating the underlying pathophysiology, thereby exacerbating the event [5].

The major limitation of the CRUSADE study is its retrospective and observational nature. In addition, the dosing of morphine was not collected and so it could not be determined how this variable might influence clinical outcomes. Meine et al. make a good effort to account for these limitations, however, by performing subgroup analyses as well as utilizing generalized estimating equations to adjust for correlations among responses. Overall, the magnitude of the study’s 57,039 subject enrollment size acquired across 443 hospitals is perhaps its greatest strength and lends significant weight to the soundness of its findings [5].

In 2010, Iakobishvili et al. published a retrospective analysis of the 30-day outcomes stratified by IV narcotic (IVN) use, defined as "primary morphine," among ACS patients, using logistic regression and a propensity score analysis in order to evaluate the safety of this treatment in this population. The data was collected from the acute coronary syndrome Israeli survey (ACSIS) 2008 database, a prospective nationwide survey conducted anonymously from March 15 to May 15, 2008, during initial hospitalization in all 26 coronary cardiac units and cardiology wards in Israel. A total of 1,758 patients were enrolled; 765 patients with ST-elevation acute coronary syndromes (STE-ACS) and 993 patients with NSTE-ACS. All patients with a final diagnosis of ACS were included [11].

Patients were stratified by their diagnosis of STE-ACS or NSTE-ACS and then further stratified by whether they received an IVN during their hospitalization course. A logistic regression model was used to estimate the multivariate adjusted odds ratio of a 30-day endpoint of death, with adjustments done for age, gender, Killip class, previous myocardial infarction, previous angina, previous congestive heart failure, renal failure, peripheral vascular disease, previous stroke, hypercholesterolemia, hypertension, diabetes mellitus, heart rate, systolic blood pressure, and propensity score for IVN use [11].

A data analysis showed that 261 STE-ACS patients (34.1%) received IVN, compared to 97 (9.8%) in the NSTE-ACS subgroup. The odds ratio for a 30-day death in IVN users was 0.40, with a 95% confidence interval of 0.14 to 1.14 and a p-value of 0.08. These results suggested that morphine use is not necessarily detrimental and may even prove beneficial in certain patients if used appropriately, directly contradicting the results of the CRUSADE study. However, because the ACSIS study took place approximately five years later, Iakobishvili et al. acknowledge that it is possible clinical practice had already been influenced by the landmark findings of the CRUSADE publication, leading to the disparity in findings. In addition, a significant difference in baseline characteristics was found in patients who received IVN and those who didn’t in the ACSIS study while no significant difference existed in the CRUSADE study. This suggests that perhaps more selective criteria for candidates receiving IVN may have already been implemented in practice. The limitations of this study are associated with its retrospective nature as well as the relatively small size of its cohort when pitted against the exponentially larger CRUSADE study [11].

In 2018, Farag et al. published a prospective, single-center study of 300 patients studying the effect of morphine use on thrombotic status, reperfusion, and infarct size in adult patients with STEMI. All patients received ticagrelor and aspirin; however, those with chest pain described by the patient as severe received intravenous morphine and ondansetron. There were no significant baseline differences between the two groups. Blood was taken and sampled, assessing for thrombotic status, in a blind fashion. In addition, electrocardiogram (ECG) and angiographic interpretation and analysis assessing for ST-segment resolution and infarct-related arterial flow were also blinded [12].

The results of the study revealed that patients who received morphine were less likely to experience ST-segment resolution before PCI (9.2 vs 31.7%, p < 0.001). Additionally, flow through the involved artery by angiography was more reduced in those that received morphine (21.6% vs. 48.8%, p=0.001). Peak troponin levels were higher in patients who received morphine (p=0.016). Patients treated with morphine exhibited enhanced platelet reactivity and impaired endogenous fibrinolysis. There was no significant difference in major adverse cardiac events and death [12].

This study is strengthened by its large sample size of 300 and blinding; however, due to the nonrandomization, there are potential confounders. Those patients that reported severe pain and received morphine may have had a worsened baseline infarction leading to decreased resolution and reperfusion. Furthermore, although morphine use appeared to be associated with a prothrombotic state and reduced reperfusion, this did not translate to concrete patient-oriented outcomes such as reinfarction, stroke, or death [12].

Many non-opioid alternatives to morphine have been considered, however they are not without their own challenges. Intravenous nitroglycerin offers even more efficient perfusion and oxygen supply than its sublingual counterpart; however, its potential for inducing severe hypotension cannot be overlooked. Nonsteroidal anti-inflammatory drugs also offer effective pain relief, but their association with increased cardiovascular events is cause for contraindication in this population [13].

The synthetic opioid fentanyl was also a promising alternative to morphine. In a 2016, randomized, double-blind, controlled trial, it had demonstrated analgesia safety and efficacy comparable to morphine [14]. However, the recent Platelet Aggregation with tiCagrelor Inhibition and FentanYl (PACIFY) trial by McEvoy et al. demonstrated that like morphine, fentanyl also has antiplatelet inhibitory properties [15]. 

Table 1 provides a synopsis of the reviewed studies.

**Table 1:** Summary of Studies

Outcome Studied	Study	Study Design	Sample Size	Number Receiving Morphine	Result
Infarct Size	Gwag et al. (2017) [8]	Prospective, nonrandomized, observational	299	96 (32.1%)	Increased
	de Waha et al. (2015) [9]	Retrospective, nonrandomized, cohort	276	123 (44.6%)	Increased
Antiplatelet Absorption Time	Hobl et al. (2014) [10]	Randomized, double-blind, placebo-controlled	24	12 (50%)	Delayed
	Kubica et al. (2016) [6]	Randomized, double-blind, placebo-controlled	70	35 (50%)	Delayed
	Parodi et al. (2015) [7]	Prospective, observational	300	95 (31.7%)	Delayed
Mortality	Meine et al. (2005) [5]	Retrospective, nonrandomized, observational	57,039	17,003 (29.8%)	Increased
	Iakobishvili et al. (2010) [11]	Retrospective, nonrandomized, cohort	1,758	358 (20.4%)	No significant difference
	Farag et al. (2018) [12]	Prospective, nonrandomized	300	218 (72.7%)	No significant difference

## Conclusions

Ever since the publication of the CRUSADE study in 2005, the appropriateness of morphine use in ACS has been hotly contested. Subsequent studies in recent years have continued to fuel concerns with findings that further characterize the negative impact that morphine may have on the pathophysiology of ACS leading up to death. Several groups have already commented on its alarming effects on infarct sizes, absorption of oral drugs, as well as overall mortality. Though the results of the ACSIS study did not mirror those of CRUSADE's, it suggests the notion that perhaps morphine would not be so deleterious or it may even be advantageous if administered in the right patient. Therefore, further research into the development of more precise criteria for IV morphine administration in ACS patients is needed.

Due to the importance of pain relief in preventing further ischemic damage in ACS, it is imperative to provide sufficiently potent options for analgesia that do not pose a further risk in driving negative outcomes. Until further randomized control trials are performed with results that clearly show negative patient-oriented outcomes, these studies should likely not change any guidelines or influence physician practice patterns.
